# Checkpoint Inhibitor in a Melanoma Patient With Polyendocrinopathy and Gangrenous Gallbladder With a Mass

**DOI:** 10.7759/cureus.8786

**Published:** 2020-06-23

**Authors:** Nayson L Fernandes, Soujanya Sodavarapu, Sukhmine Nedopil, Nikita Mohapatra, Dinesh Vyas

**Affiliations:** 1 Surgery, California Northstate University, Elk Grove, USA; 2 Internal Medicine, San Joaquin General Hospital, French Camp, USA; 3 Surgery, San Joaquin General Hospital, French Camp, USA; 4 Bioengineering, University of California, Berkeley, USA

**Keywords:** pembrolizumab, keytruda, checkpoint inhibitors, malignant melanoma, polyendocrinopathy, cholecystitis, transaminitis, cholecystectomy, hypophysitis, adrenal insufficiency

## Abstract

Checkpoint inhibitors are introduced as a therapy for clinical use for various cancers, and clinicians are documenting new adverse effects. This is the first case report to the best of our knowledge of a patient on checkpoint inhibitor presenting with both polyendocrinopathy and gangrenous gallbladder disease with a mass negative for malignancy.71-year-old man presented four years after his initial diagnosis of stage IV, unresectable, non-ulcerated, acryl, lentiginous malignant melanoma. On presentation, he had gangrenous cholecystitis and was treated with laparoscopic cholecystectomy. Incidentally, the patient was diagnosed two years ago with hypothyroidism, hypophysitis, secondary adrenal insufficiency, and pneumonitis, each suspected to be secondary to treatment with pembrolizumab (Keytruda), a monoclonal anti-programmed cell death-1 antibody. He presented to the emergency department for a gallbladder attack and underwent successful laparoscopic cholecystectomy. The intra-operative finding on opening the specimen was an unusual looking exophytic mass but was negative for malignancy on pathology report and reported as gangrenous cholecystitis. His clinical condition before and after surgery was complicated by worsening comorbidities thought to be secondary to pembrolizumab therapy, which required acute care hospitalizations in the weeks before and after his presentation with cholecystitis. The patient had a few admissions from other co-morbidities post-surgery and was doing better. Immunotherapy with pembrolizumab may have secondary and tertiary effects with unusual presentations that are difficult to interpret for the primary oncology team and even tougher to do for community physicians who may subsequently encounter these patients. The relationship of this patient’s comorbidities with immune-related adverse events was not apparent until record requests were conducted after surgery and are still not entirely clear after a literature review. More data is needed to guide decision algorithms and to predict which patients may experience these effects.

## Introduction

Immunotherapy with checkpoint inhibitors has revolutionized the care of advanced melanoma. Patients with inoperable metastatic melanoma have a chance to not only slow cancer progression but to stop and reverse it, with fewer side effects as compared to conventional chemotherapy [1-2]. These promises have led to a rapid increase in the use of checkpoint inhibitors and increased longevity for terminally ill cancer patients. Side effects, while rare, can be life-threatening and require early recognition and prompt interventions to ensure survival. We describe a case of a 71-year-old man with inoperable metastatic melanoma on pembrolizumab treatment who presented to a community hospital not involved with his oncology care with acute acalculous cholecystitis superimposed over chronic pneumonitis and polyendocrinopathy secondary to pembrolizumab treatment.

## Case presentation

**Figure 1:**
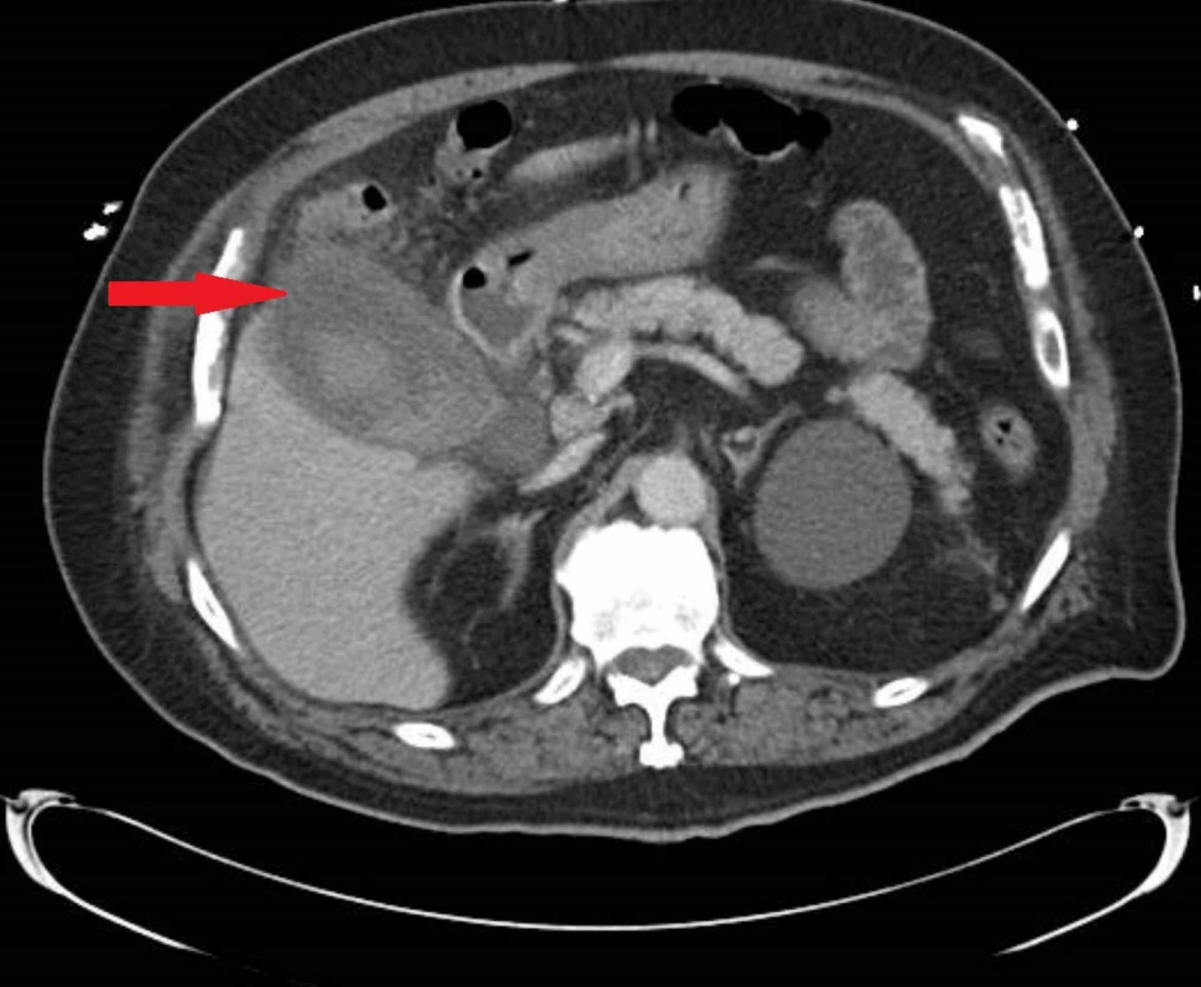
Computed tomography of the abdomen with contrast showing distended gallbladder with surrounding inflammatory changes

A 71-year-old man, with a history of hypertension, diabetes, gout, hypothyroidism from radioactive ablation of Grave’s disease, atrial fibrillation, and metastatic melanoma, complained of fever, abdominal pain associated with nausea, and non-bloody vomitus since two days. The patient has a history of wide lesion excision with a split-thickness graft a year prior, treated with pembrolizumab (cycle 11), and side effects secondary to pembrolizumab, including dermatitis, pneumonitis, hypophysitis, and adrenal insufficiency treated with high dose steroids. At presentation, the patient was febrile, tachycardic at 90 beats/min, and tachypneic at 23/minute. The physical exam was significant tenderness to palpation in the right lower quadrant, with a negative Murphy’s sign. Blood workup showed elevated liver function tests with alkaline phosphatase 492.0 IU/L, alanine transaminase 146 IU/L, aspartate transaminase 125 IU/L, and total bilirubin 4.8 mg/dL. Ultrasound of the abdomen showed an abnormal thick-walled gallbladder with pericholecystic fluid, internal debris/ sludge, and common bile duct (CBD) diameter of 6 mm. Ultrasound done four weeks prior was unremarkable. Computed tomography (CT) of the abdomen with contrast showed distended gallbladder with internal debris, pericholecystic fluid, and wall thickening highly suggestive of cholecystitis (Figure 1). Magnetic resonance imaging (MRI) abdomen showed acute cholecystitis, nobiliary dilatation, or choledocholithiasis. Subsequently, endoscopic retrograde cholangiopancreatography (ERCP) was done with biliary sphincterotomy, and multiple balloon sweeps of the CBD showed a normal appearance. The Cholangiogram at the end of ERCP was normal as well. The patient was diagnosed with acute acalculous cholecystitis and laparoscopic cholecystectomy was performed without complications.

Microscopic analysis of the gallbladder specimen revealed acute and chronic inflammatory cells, necrotizing inflammation, and gangrenous necrosis. Blood cultures have been negative. No dysplasia or malignancy was identified. Postoperatively, the patient was hemodynamically stable and was discharged. The patient had shock-like episodes over the next month, requiring multiple hospitalizations. Workup during these hospitalizations was consistent with pneumonitis and continued transaminitis that his primary oncology team suspected to be melanoma metastases. The patient’s hemodynamic instability was considered to be secondary to polyendocrinopathy and prompted his oncology team to hold pembrolizumab.

Per outside records, the patient first experienced dermatitis, requiring high-dose steroids after eight cycles (five months) on pembrolizumab, which was later held. During the taper down from prednisone treatment, the patient demonstrated adrenal insufficiency consistent with hypopituitarism, was transitioned to hydrocortisone, and his symptoms stabilized. He was re-initiated on pembrolizumab after a five-month period due to disease progression and subsequently completed an additional three cycles over six weeks (11 cycles total, over 11 months). Less than one month after his eleventh infusion, he developed hypoxic acute respiratory failure and septic shock followed by acalculous cholecystitis two weeks later. Records from subsequent hospitalizations suggest continued pneumonitis and transaminitis of unknown etiology. After these events, his oncologists decided to hold pembrolizumab treatment for the second time.

## Discussion

**Figure 2:**
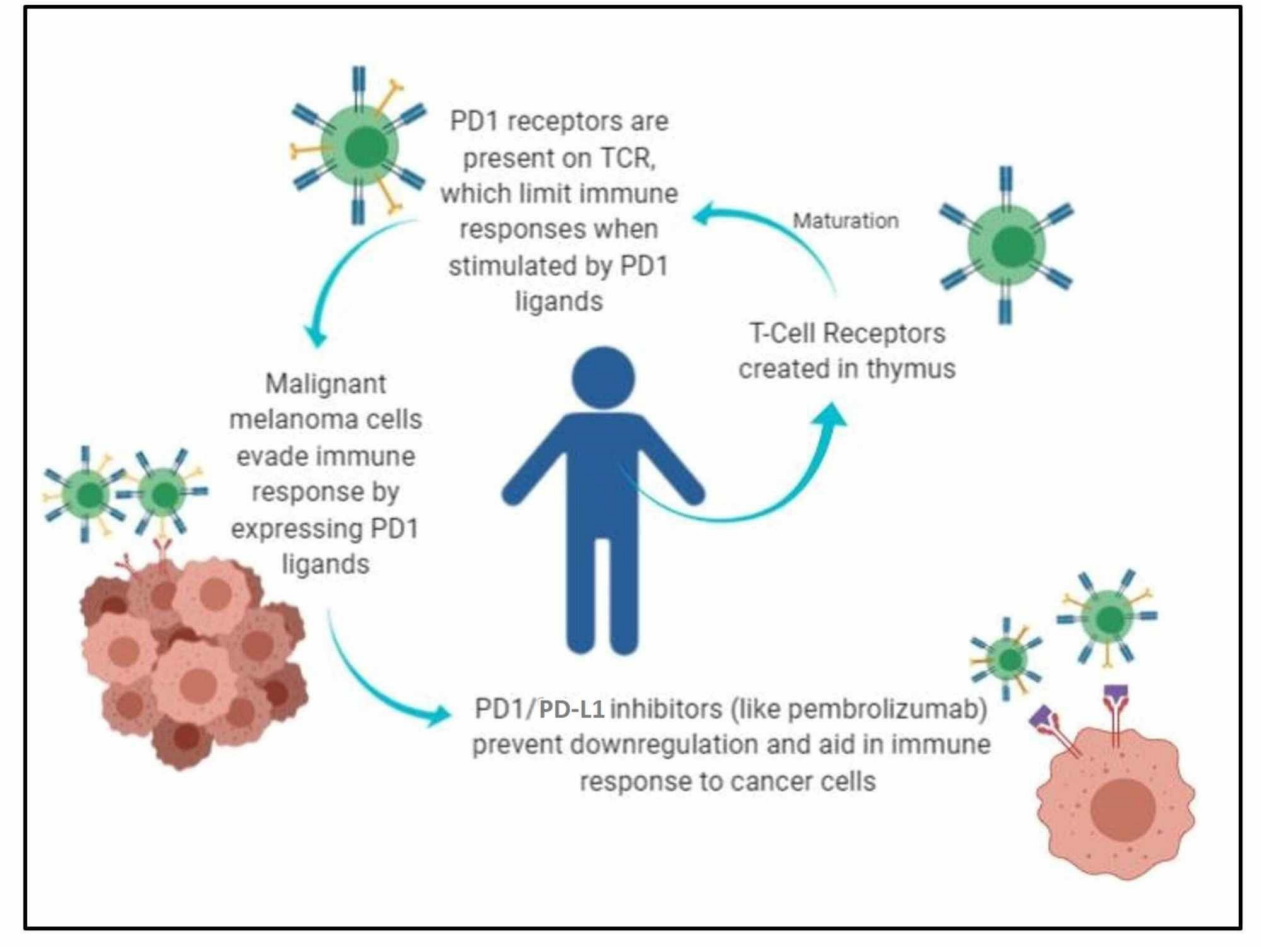
Mechanism of action of PDL1 inhibitors on immune cells PD1: programmed cell death protein 1

Immunotherapy with checkpoint inhibitors has revolutionized the care of advanced melanoma. The programmed death 1 (PD-1) pathway belongs to a class of checkpoint inhibitors that utilize B7/CD28 co-stimulatory receptors to modulate immune responses [3]. Malignant melanoma cells can express PD-L1 and mimic the antigenic milieu of their surroundings, thus evading an immune response [4]. PD-1 and PD-L1 inhibitors prevent this downregulation, thus aiding the immune detection and response to cancer cells (Figure 2) [5].

**Figure 3:**
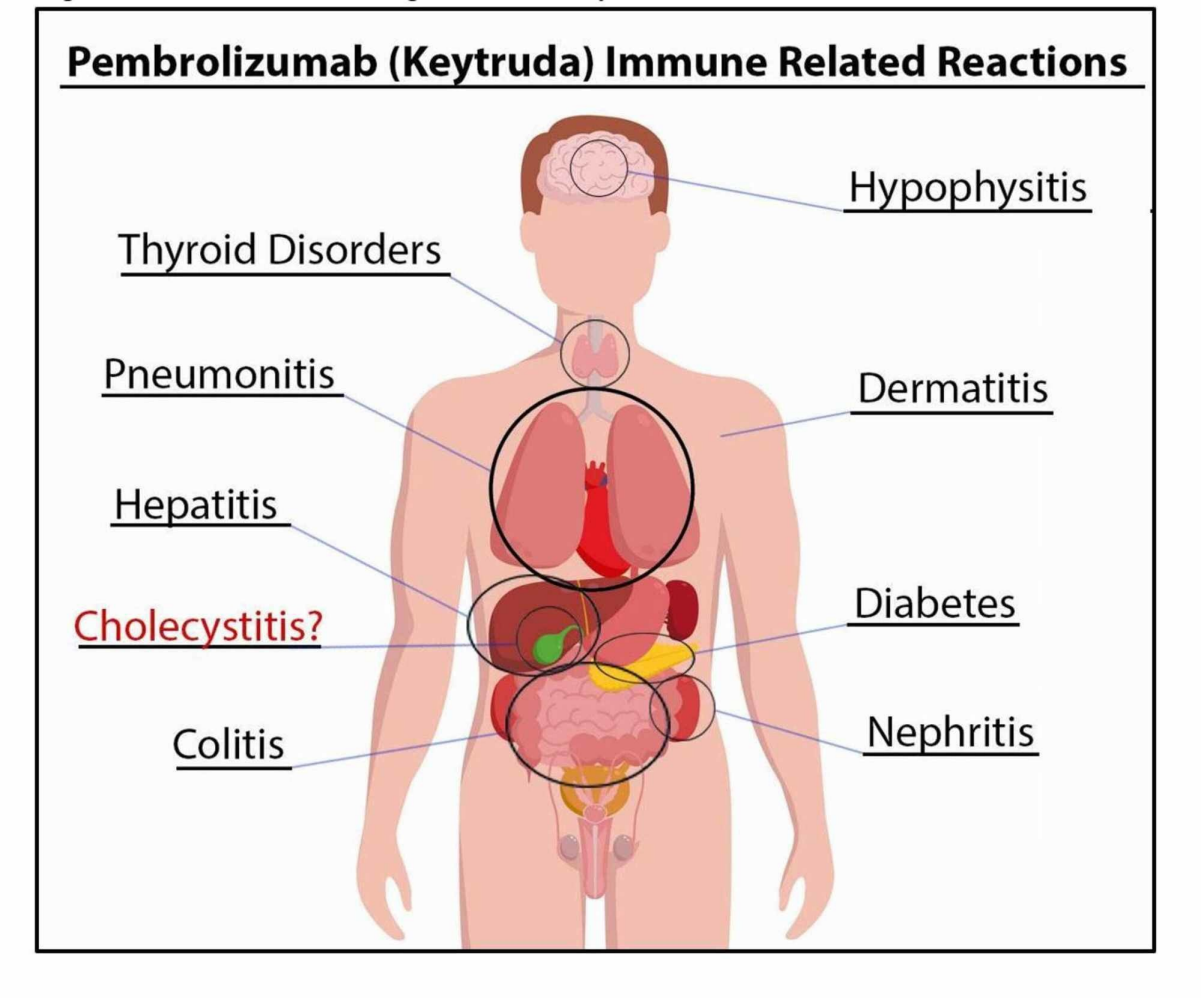
Potential sites of organs affected by PD1 inhibitors PD1: programmed cell death protein 1

PD-1 checkpoint signaling inhibition is found to increase longevity and be more tolerable compared to conventional chemotherapy for the treatment of certain cancers [5]. Current data suggest that PD-1 inhibitors are effective treatments for metastatic melanoma [3,6-10]. Though preliminary data suggest that improved survival is seen in patients whose tumor cells show high PD-L1 expression [11-12], the relationship between the side effects and effectiveness of PD-1 inhibition is still unclear. Figure 3 (original) depicts these immune-related effects. One odd feature of PD-1 inhibition is that side effects may present seemingly with immune-related adverse events, many months or years after treatment [13].

A recent review suggested that additional gastrointestinal effects, such as vanishing bile duct syndrome and acute liver failure, may also be related to pembrolizumab treatment [14]. Cholecystitis due to other PD-1 inhibitors like nivolumab and avelumab treatment have been reported [15-16]. Another case series reported cholecystitis in 0.4% of patients after receiving immune checkpoint inhibitor treatment but have not mentioned which particular drug was used [17]. However, no cases of cholecystitis subsequent to pembrolizumab treatment have been published.

Here, we report a case of cholecystitis in a patient with malignant melanoma and several comorbidities likely secondary to pembrolizumab treatment. Determining the origin of new symptoms in such a patient is difficult; symptoms may be the result of cancer progression, secondary effects of pembrolizumab, side-effects of the treatments for secondary effects of pembrolizumab, or unrelated new disease. Differentiating the origin of symptoms is even more challenging when the patient presents to physicians at a community hospital who are unaware or unable to fully appreciate the complex past medical history of such patients on an emergent basis.

**Figure 4:**
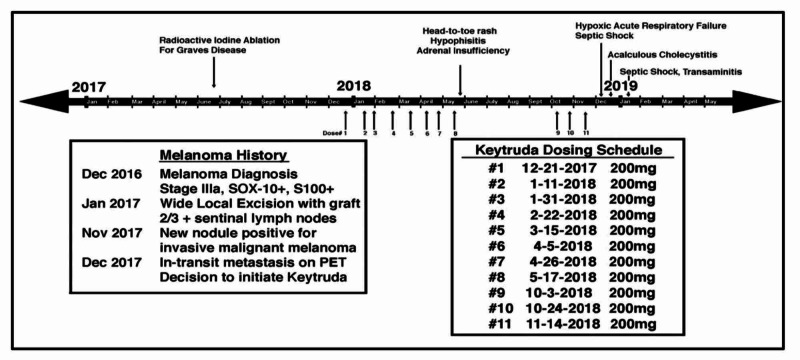
Timeline of patient history and treatment with Keytruda

An interesting aspect of this case is the order of events and the time frame it took for each of these patient’s side effects to be teased apart as primary, secondary, or unrelated causes of pembrolizumab treatment. Our patient presented with multiple endocrine abnormalities requiring management, including poorly controlled diabetes mellitus, hypothyroidism, and adrenal insufficiency, in addition to his primary signs and symptoms of acute cholecystitis. Record requests from the hospital managing his oncology care, after the surgery was conducted, revealed that most of these comorbidities were related to the patient’s pembrolizumab treatment. A timeline of this patient’s pembrolizumab infusions and the disease course is depicted in Figure 4.

Thus, the order of suspected immune-related events in our patient was dermatitis, followed by hypophysitis, secondary adrenal insufficiency, pneumonitis, cholecystitis, and, finally, transaminitis. Prior cases have revealed similar comorbidities attributed to pembrolizumab treatment, with one similar case of polyendocrinopathy showing thyroiditis preceding adrenal insufficiency [18]. Our patient had hypothyroidism prior to his treatment with pembrolizumab due to radioactive ablation for Graves disease. This raises an important question - is there a relationship between pre-existing autoimmune disease and the development of autoimmune-related side-effects of pembrolizumab treatment, as patients with prior autoimmune diseases are excluded in many trials.

Some evidence suggests that patients prone to adverse effects related to immunotherapy treatment may also be among the highest responders to treatment [19-20]. While this news is positive, we were unable to find published data regarding the relationship of autoimmune side effects of pembrolizumab with previous autoimmune disease burden or efficacy of response.

In this case, the first immunotherapy-related adverse event noted on record review is dermatitis that was at first attributed to amoxicillin treatment for a dental procedure. Next, adrenal insufficiency was thought to be secondary to steroid withdrawal for dermatitis and was not attributed to hypophysitis for nearly two months. Our patient’s multiple episodes of shock prior to and after cholecystectomy may have been preventable with closer titration of steroid treatments. However, the precise hypothalamic pituitary adrenal (HPA) axis dysfunction and an appropriate treatment algorithm were not elucidated due to a delay in recognizing symptoms. More definitive diagnostic testing with MRI is also missing due to the patient’s acute delirium at the time and positive response to steroid treatment. Of note here, the patient had oncology care at an academic hospital that was in a different county than his home community hospital in which he presented for several immune-related adverse events that have added to the complexity of our patient’s care.

## Conclusions

Treatment with pembrolizumab may result in life-threatening, immune-related comorbidities that may be hard to detect and treat, particularly for community physicians who are not part of the patient’s oncology care team. Effective acute care of such patients will require clinicians to be mindful of a broad range of side-effects and their presentations. The relationship between pre-existing auto-immune disease and treatment efficacy is unclear. Severe immune-related adverse events with these treatments need to be considered. More data are needed to predict responders and to guide treatment algorithms in those with adverse immune reactions. Physicians and all members of an immunotherapy patient’s care team must be educated and prepared to address novel side effects and the complex care required to treat these patients.
